# Beyond detection rate: understanding the vigilance decrement using signal detection theory

**DOI:** 10.3389/fcogn.2024.1505046

**Published:** 2025-01-17

**Authors:** Henri Etel Skinner, Barry Giesbrecht

**Affiliations:** ^1^Department of Psychological & Brain Sciences, University of California, Santa Barbara, Santa Barbara, CA, United States; ^2^Institute for Collaborative Biotechnologies, University of California, Santa Barbara, Santa Barbara, CA, United States

**Keywords:** vigilance, signal detection theory, sustained attention, performance, inattention

## Abstract

The vigilance decrement has been classically characterized as the decline in performance across time as individuals continuously attend to a task. Errors during these periods of degraded performance are often collectively characterized as failures of attention. Methodologically, the classic characterization of the vigilance decrement relies upon declines in detection rate, a binary measure that is unable to characterize performance beyond a single dimension. Theoretically, using a single construct, such as attention, to describe impaired performance obscures what is likely a range of behaviors. This is a critical issue for the study of vigilance because detection rate can be impacted both by changes in sensitivity and decision criterion. Commonly used tasks do not allow for the reliable computation of these metrics because they elicit a low number of false alarms or because they introduce confounding response demands. To address these shortcomings, we propose the use of a paradigm amenable to the application of the signal detection framework, which permits the reliable and isolated investigation of the vigilance decrement across multiple measures.

## 1 Introduction

Sustained attention is the continuous delegation of information processing resources across time to a task. One pervasive sustained attention phenomenon is present under conditions requiring vigilance, characterized by the decline in performance across time when continuously monitoring for rare targets. This vigilance decrement was initially reported in the Mackworth Clock Task where detection for rarely occurring discrepancies in clock hand movements dropped over time (Mackworth, 1948). Since that seminal finding, decades of study of the vigilance decrement has addressed core issues in the theoretical understanding of sustained attention and provided diagnostic tools for critically impaired sustained attention in clinical populations (Pattyn et al., 2008; Rueckert and Grafman, 1996; Smit et al., 2004).

Current methods in vigilance tasks largely rely upon single measures like detection rate and performance is often characterized by a generic construct of attention. Unfortunately, these approaches do not allow for the complete characterization of performance because they obscure processes associated with other contributing factors like response bias. Here, we describe signal detection theory and how it addresses current methodological and theoretical constraints on the study of the vigilance decrement imposed by relying on detection rates alone. We then describe a paradigm amenable to the application of signal detection theory and optimized to study the vigilance decrement. This paradigm addresses current methodological and theoretical constraints by providing measures that more fully characterize behavior and allow for interpretations beyond a generic construct. Lastly, we discuss the implications of this perspective for the broader study of sustained attention, addressing limitations of the proposed paradigm and highlighting the strengths of other current methods.

## 2 Signal detection theory: a primer

Signal detection theory (SDT) was proposed for the study of psychophysical constraints on the senses in simple decision-making paradigms (Fechner, 1860/1966; Green and Swets, 1966; Layher et al., 2020; Macmillan and Creelman, 2004; Stanislaw and Todorov, 1999; Wickens, 2001). This perspective provides a brief introduction to key constructs and their application to the topic of sustained attention (for more detailed explanations see Wickens, 2001).

**Figure 1:**
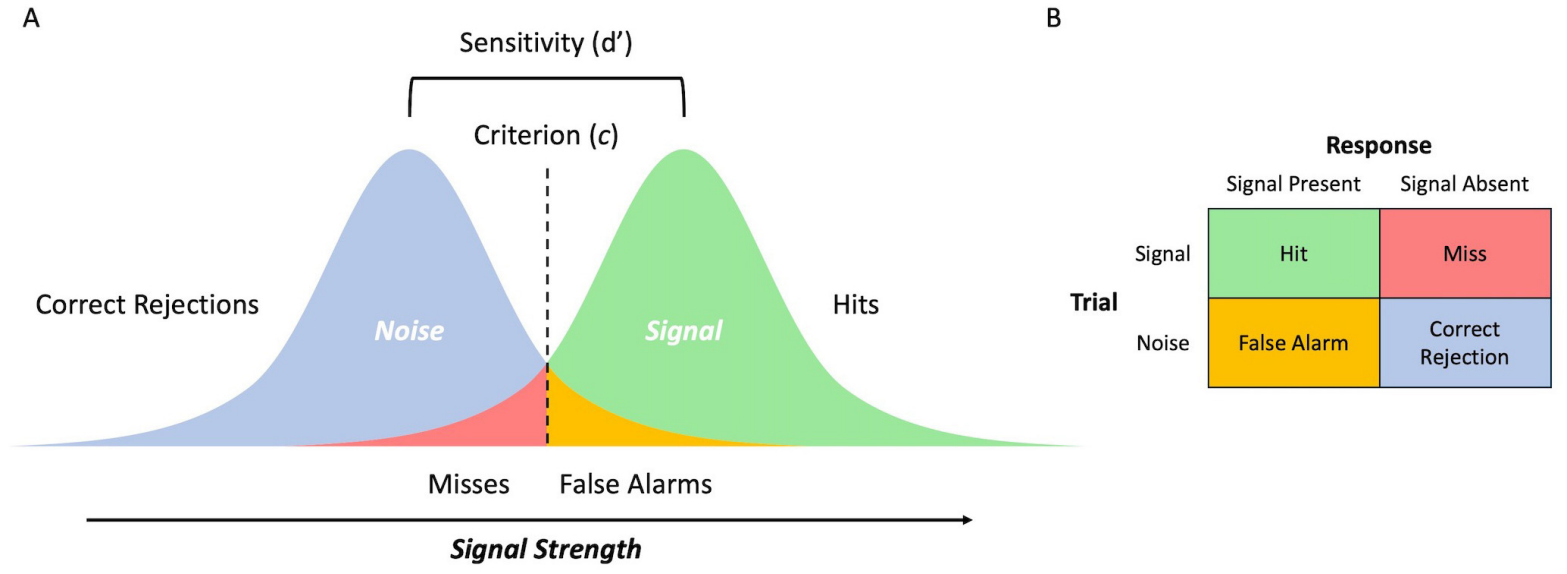
Overview of the signal detection framework. **(A)** Signal and noise distributions lie along a signal strength axis, with distribution height indicating the relative probability of that stimulus type at that level of signal strength. Sensitivity is the distance between distribution means. Criterion is the minimal level of signal strength at which subjects respond “signal present”. **(B)** Trial identity and subject response create the classifications: Hit, Miss, False Alarm, and Correct Rejection. Based on Thomson et al. (2016). Framework from Fechner (1860/1966), Green and Swets (1966), Layher et al. (2020), Macmillan and Creelman (2004), Stanislaw and Todorov (1999), Wickens (2001).

Signal detection theory provides a framework for understanding decision-making processes in uncertain conditions. The simplest conception is a binary decision of whether a signal is present or absent. Information for this decision is placed across a single dimension of evidence with independent distributions characterizing the probability of the signal being present (signal) or absent (noise) at different levels of signal strength (Figure 1). Signal and noise distributions each have additional noise, depicted as a spread from distribution means. A decision criterion is placed along the signal strength dimension. If, on a given trial, the evidence exceeds the criterion, the observer would respond signal present; if not, they would respond signal absent. Signal present responses are coded hits on signal trials and false alarms on noise trials. Signal absent responses are coded misses on signal trials and correct rejections on noise trials.

Several parameters classify performance within this framework. The first parameter is the distance (d') between the means of the signal and noise distributions, from now on described as sensitivity. The second is the placement of the decision criterion (*c*). The widths and probabilities of the distributions are other key parameters, but will be assumed to be held constant in this perspective.

The strength of signal detection theory is the ability to capture various sources that yield changes in performance. Changes in detection rate could occur from changes in sensitivity (e.g., detection rate falling as d' shrinks) and changes in criterion (e.g., detection rate falling as c shifts rightward). Therefore, studies utilizing detection rate require an account of both sensitivity and criterion in order to more fully describe behavioral phenomena. Signal detection theory has been successfully applied to fields beyond psychophysics such as memory, expanding basic and applied memory research to include new insights such as criterion shifting (Layher et al., 2020; Wixted, 2020).

## 3 Methodological and theoretical constraints

Despite the decades-long investigation of the vigilance decrement, there are persistent methodological and theoretical constraints that have limited advances in understanding the vigilance decrement.

### 3.1 Detection rate alone is not enough

Within sustained attention research, performance is often intuitively grouped into one of two categories, success or failure. This dichotomization of behavior has become integral to experimental paradigms designed around single, binary measurements of behavior. Accounts of the vigilance decrement have primarily focused upon the observed decline in detection rate in tasks where subjects monitor for rarely occurring targets (Fisk and Schneider, 1981; Galinsky et al., 1993; Grier et al., 2003; Helton and Warm, 2008; Mackworth, 1948; O'Connell et al., 2009). The main concern with relying upon detection rate is this method only allows for the interpretation of behavior across a single dimension, a problem that persists even after averaging. The sole reliance upon detection rate therefore risks conflation of different ongoing behaviors.

Even while many accounts of the vigilance decrement have primarily relied upon the observed decline in detection rate, the vigilance decrement has been persistently equated to a decline in sensitivity (See et al., 1995). This yields a potential problem because tasks that rely upon detection rate alone cannot verify a sensitivity-only interpretation of the vigilance decrement because sensitivity cannot be measured separately from criterion (Thomson et al., 2016). Therefore, in paradigms solely relying upon detection rate, is unclear if the changes in observed behavior result from changes in attention or, instead, shifts of criterion (Thomson et al., 2016). This highlights the methodological shortcomings of relying upon single measures of performance because it cannot discriminate between different constructs.

Detection rate cannot stand alone as a measure of performance because it can be impacted by multiple sources (Thomson et al., 2016). Vigilance research has identified paradigms that yield changes in strategy and attention both separately and in tandem (Berardi et al., 2001; Broadbent and Gregory, 1965; Nuechterlein et al., 1983). Therefore, the study of the vigilance decrement cannot be solely reliant upon a single measure because behaviors related to different cognitive processes may be obscured.

### 3.2 Theoretical dichotomies encourage the use of generic constructs

Changes in behavior classified on detection rate alone are treated as homogenous in nature or source and behaviors are readily assigned as successes or failures within a singular theoretical construct (e.g., “attention” or “vigilance”). Singular constructs are generic terms that broadly characterize observed behavior. These generic constructs can be useful and are validated by individual differences and clinical studies that report on single constructs of inattention (Broadbent et al., 1982; Robertson et al., 1997; Smilek et al., 2010). However, this dichotomization of behavior into successes and failures risks the collapse of multidimensional phenomena into unidimensional constructs.

The use of generic constructs is a potential issue for vigilance research because multiple cognitive processes can contribute to sustained attention performance in commonly used tasks reported in the literature (Thomson et al., 2015). While failures in performance can occur due to inattention (Fraulini et al., 2017), they can also occur independently, or alongside, changes in attention due to non-optimal performance strategies (Parasuraman, 1979; Berardi et al., 2001; McCarley and Yamani, 2021). Speed-accuracy trade-offs and incorrectly placed criterion thresholds can introduce changes to performance even when attention is stable (Dang et al., 2018; Broadbent and Gregory, 1965). Some phenomena, such as mind wandering, do not have a one-to-one relationship with attention, as mind wandering can involve both on- and off- task thought (Smallwood et al., 2004). Lapses in attention have also been related to other cognitive processes such as learning (Decker et al., 2023). Generic constructs based on the dichotomization of behavior can lead to a loss of information and this loss of information obscures understanding of the vigilance decrement.

### 3.3 Method limits theory

Two related issues that limit our understanding of the vigilance decrement have now been identified. Methodological approaches that emphasize single, binary measures like detection rate can only be interpreted across a single dimension and can therefore only inform unitary constructs of cognition. As a result, current methodology is insufficient to expand beyond unitary constructs and theories remain constrained by them. These constraints limit a holistic account of factors influencing performance because they can obscure different underlying cognitive processes. These two issues combine such that single measures of performance cannot characterize the nature of vigilance beyond a single label—a unidimensional measure cannot fully describe a multidimensional phenomenon. While the signal detection framework is well suited for the incorporation of multiple measures of performance to characterize vigilance, a methodological approach is needed that allows for the computation of signal detection measures within a single paradigm.

## 4 Addressing methodological constraints

### 4.1 Current methods are not readily amenable to SDT

The solution to these methodological and theoretical limitations requires task designs allowing for reliable computation of signal detection metrics. In classic vigilance tasks, subjects are typically required to attend for rare targets over extended periods of time (Mackworth, 1948). These vigils are effective at yielding the vigilance decrement, but have low response rates, which can yield low false alarm rates (Galinsky et al., 1993; Grier et al., 2003). Importantly, a decline in sensitivity results in both the decrease in detection rate and the increase in false alarm rate. In contrast, a conservative (rightward) shift of criterion also yields a decrease in detection rate, but a decrease in false alarm rate. The vigilance decrement has largely been characterized by a decline in detection rate across time, which could therefore be caused by a decline in sensitivity or a conservative shift of criterion. Critically, if false alarm rates are too low or at zero at the start of a vigil, conservative shifts of criterion become indistinguishable from declines in sensitivity due to a floor effect (Thomson et al., 2016). Therefore, a major limitation of current vigilance research is the use of tasks that elicit low false alarm rates because they are unable to accurately discriminate between changes in sensitivity and criterion during vigils.

### 4.2 A promising vigilance paradigm amenable to SDT

**Figure 2:**
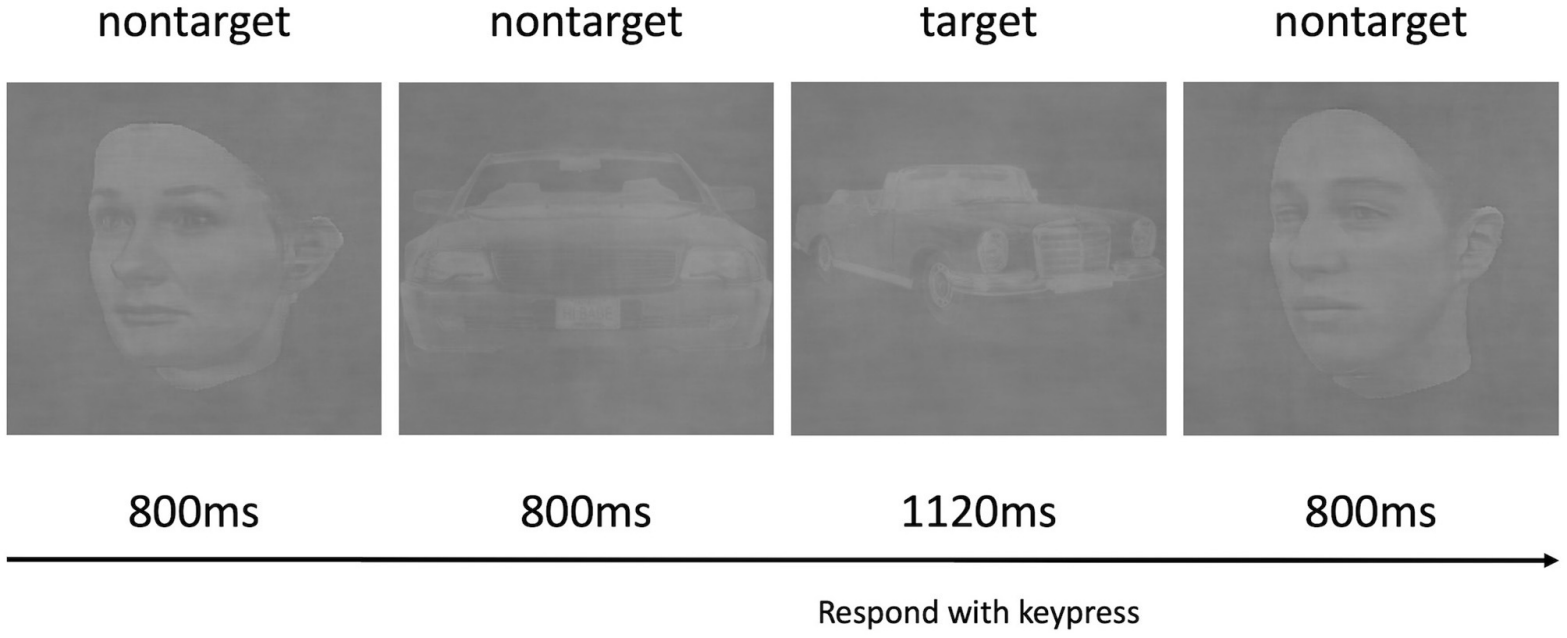
An example trial sequence of the Continuous Temporal Expectancy Task. Images appear continuously for brief periods of time (e.g., 800 ms, 90% of images) over blocks of several minutes. Subjects are instructed to only respond to long duration (e.g., 1,120 ms, 10% of images) images. Depicted stimulus durations are the same as in O'Connell et al. (2009), but actual stimulus durations may vary (see Galinsky et al., 1993).

Here, and in prior research, it has become clear that the study of vigilance requires a task that consistently yields the vigilance decrement while avoiding low false alarm rates (Berardi et al., 2001; Grier et al., 2003; Parasuraman, 1979; Thomson et al., 2016). One promising paradigm is the Continuous Temporal Expectancy Task (CTET, Gray et al., 2015; O'Connell et al., 2009) in which a continuous stream of images appears one at a time in a centralized display (Figure 2). Most (e.g., 90%) of the images appear for a brief standard period of time (800 ms). The remaining minority (e.g., 10%) of the images appear for a slightly longer period of time (1,120 ms) (O'Connell et al., 2009). Subjects monitor the stream of images and respond only when an image has appeared for a longer duration. Studies using the CTET report a rapid and robust vigilance decrement emerging within 3 minutes of continuous task performance (O'Connell et al., 2009).

Much like other vigilance tasks, studies using the CTET have only reported declining detection rates as an indicator of performance because false alarm rates in this task are low. Importantly however, this task is unique from other low target prevalence tasks because the vigilance decrement can be repeatedly induced in relatively short periods of time (~3 vs. 120 min) (Mackworth, 1948; O'Connell et al., 2009). Therefore, task manipulations yielding higher false alarm rates (such as shifting initial response bias) could be especially effective because within a realistic behavioral testing session the vigilance decrement could be induced multiple times, increasing the reliability and sensitivity of signal detection measures. While the use of criterion manipulations to bolster signal detection computation has not been reported for the CTET, modifications of prevalence, task instructions, and reward structure that shift criteria have been employed in the study of vigilance and other fields (Aminoff et al., 2015; Baddeley and Colquhoun, 1969; Layher et al., 2020; Wolfe et al., 2013).

The CTET design also allows for the isolation of mechanisms involved in the vigilance decrement from other cognitive functions. Low response rates avoid response inhibition demands, an issue for tasks with high response rates that measure performance on the ability to withhold responses to rarely occurring targets (Carter et al., 2013; Stevenson et al., 2011). The ability of the CTET to evoke a vigilance decrement at such a rapid pace and with low response rates is unique. In particular, it is distinguished from existing paradigms (SART, gradCPT) that have been modified to elicit low response rates because these modifications do not yield a robust vigilance decrement (Carter et al., 2013; Jun et al., 2019; Jun and Lee, 2021). The subtle target-defining feature, only identified by a difference in stimulus duration, minimizes confounds from post-error processing which are common to tasks with salient target-defining features (Cheyne et al., 2009). Lastly, the CTET minimizes speed-accuracy trade-offs, as there are no built-in imperatives to respond quickly (Dang et al., 2018; Head and Helton, 2014).

The isolation of the vigilance decrement within the CTET, combined with the application of signal detection theory, addresses current theoretical and methodological limitations reviewed here. The CTET is amenable to criterion manipulations, potentially allowing for the rich assessment of task behavior across detection rate, false alarm rate, sensitivity, and criterion. This revised methodology, from a single metric to a set of metrics, facilitates the measurement of the respective contributors to the vigilance decrement. Ultimately, this paradigm addresses theoretical constraints in investigating vigilance by allowing for the characterization of behavior beyond a generic construct and instead across multiple contributing factors to the vigilance decrement.

While the CTET addresses existing methodological and theoretical limitations, there are three noteworthy caveats to this approach. First, a low target rate does not allow for high-temporal resolution measurements of performance across time. Concurrently, low response rates do not provide reliable measurements of noisy variables like response time. Furthermore, neither the signal detection framework nor CTET demands have a clear interpretation of response time, even though response time measures are also essential to understanding sustained attention (Cheyne et al., 2009; deBettencourt et al., 2019; Esterman et al., 2013). Despite these considerations, the proposed paradigm captures multiple aspects of performance, which will play a critical role in expanding our understanding of the vigilance decrement.

## 5 Discussion

Vigilance research has been limited by classifying performance within a dichotomy of success and failures even though the source and nature of these outcomes is not homogeneous. Vigilance theories are constrained by the use of single measures like detection rate. Current methods limit theory, as current paradigms do not allow for characterizations of performance outside of unitary constructs. The CTET appears one promising next step in the investigation of vigilance as it allows for a more diverse account of the cognitive mechanisms involved in performance changes across time. However, a concrete understanding of the vigilance decrement requires both a close-up view of its properties in isolation as well as in the field of sustained attention as a whole.

### 5.1 Other approaches

Where studies of the vigilance decrement utilize tasks with low response rates, other sustained attention studies utilize tasks where subjects respond to a majority of trials and withhold responses to a minority of trials. One prominent task is the Sustained Attention to Response Task (SART, Robertson et al., 1997) in which subjects view a continuous stream of stimuli (e.g., a set of numbers, 1-9) appearing one at a time. Subjects respond to a majority of stimuli (e.g., all but the number three) and withhold responding to rarely occurring targets (e.g., the number three). Similarly, the gradual-Continuous Performance Task (grad-CPT) requires subjects to respond to frequently occurring standard (i.e., nontarget) images (e.g., cities) and withhold responding to rarely occurring target images (e.g., mountains), with images gradually phasing from one to the next (Esterman et al., 2013; Rosenberg et al., 2013). In these tasks, performance is calculated primarily on the ability to withhold responding to rarely occurring targets and the response time to frequently occurring standards.

These tasks are similar to the CTET in that they minimize the amount of time needed to elicit drops in performance (8 min). Unlike the CTET, they are sensitive to fluctuations in performance at higher temporal resolutions by obtaining frequent probing of accuracy and response time (Esterman et al., 2013). These methods are compatible with real-time triggering procedures to probe attention at different levels of task engagement (deBettencourt et al., 2019; Shelat et al., 2024). These tasks even yield enough responses to generate signal detection measures (Bedi et al., 2023, 2024; Esterman et al., 2014). Despite these strengths and critical to the study of vigilance, researchers have argued performance on these tasks is dependent on changes in cognitive mechanisms besides vigilance, in particular inhibitory control (Carter et al., 2013; Stevenson et al., 2011). Therefore, while the unique response requirements of these tasks provide information about continuous fluctuations in attention, they are not well suited to understand the vigilance decrement independently of inhibitory control.

### 5.2 Future directions

We propose the development of task paradigms utilizing the CTET for the characterization of the vigilance decrement across signal detection parameters. There are straightforward modifications to signal detection parameters such as the means, widths (variances), and relative probabilities of signal and noise distributions (i.e., prevalence) within this task to investigate the impact of specific changes to task properties. Novel application of signal detection theory also provides a richer characterization of inattention across individual differences and clinical populations, as documented failures of attention are likely both quantitatively and qualitatively unique (Forster and Lavie, 2016; Osmon et al., 2018; Rosenberg et al., 2017).

## 6 Conclusion

When the investigation concerns the nature and source of the vigilance decrement, a novel methodological approach is needed that is sensitive to a range of factors that contribute to performance. Classic vigilance tasks are limited to the assessment of performance based on detection rates alone. Other sustained attention tasks, such as the SART, are well suited to study sustained attention in a continuous fashion, but are confounded by other demands such as response inhibition. Our approach is aimed at resolving the methodological problem of persistent low false alarm rates in classic vigilance tasks to permit the characterization of vigilance within the signal detection framework and allow for the generation and refinement of multidimensional theories of vigilance performance.

## Data Availability

The original contributions presented in the study are included in the article, further inquiries can be directed to the corresponding author.
